# Effects of Pulmonary Vein Isolation on Left Atrial Hemodynamic and Mechanical Function: Insights from Advanced and Emerging Echocardiographic Parameters in Responders and Non-Responders

**DOI:** 10.3390/jcdd13100483

**Published:** 2026-09-25

**Authors:** Jonasz Kozielski, Monika Olczyk, Marek Szołkiewicz

**Affiliations:** Department of Cardiology and Interventional Angiology, Kashubian Center for Heart and Vascular Diseases, Pomeranian Hospitals, 84-200 Wejherowo, Poland

**Keywords:** atrial fibrillation, pulmonary vein isolation, catheter ablation, left atrium

## Abstract

Background: Atrial fibrillation (AF) is associated with progressive remodeling of the left atrium (LA). Pulmonary vein isolation (PVI) is an established rhythm-control strategy; however, its long-term impact on LA mechanical and hemodynamic function remains insufficiently defined. Objectives: Our objective was to assess long-term changes in LA structure, mechanics, and hemodynamics after PVI, and to compare remodeling patterns between responders and non-responders. Methods: In this prospective observational study, 51 patients undergoing PVI were enrolled. Transthoracic echocardiography was performed before ablation and after 12 months. LA assessment included volumetric parameters, speckle-tracking-derived strain, and advanced functional indices (LA ejection fraction [LAEF], LA emptying fractions, LA function index [LAFI], and LA stiffness index [LAS-si]). Results: Forty-two patients (mean age 61 ± 9 years, 59.6% male) were included; a total of 32 (76.2%) were classified as responders and 10 (23.8%) as non-responders. Longitudinal differences between groups were tested using a Group × Time mixed-design ANOVA with Benjamini–Hochberg correction across the 19 echocardiographic parameters assessed. Non-responders showed a numerically greater decline in peak left atrial reservoir strain (PALS) than responders (95% CI 2.28–10.44; nominal *p* = 0.003), but this did not remain significant after correction for multiple comparisons (FDR-adjusted *p* = 0.06) and is therefore considered hypothesis-generating. Directionally consistent, non-significant trends toward greater adverse change among non-responders were also observed for LA volume index, LA ejection fraction, and LA stiffness index (all nominal *p* 0.07–0.13; all FDR-adjusted *p* ≥ 0.32). No parameter retained significance after correction for multiple comparisons. ***Conclusions:*** In this hypothesis-generating, exploratory study, recurrent arrhythmia after PVI was associated with numerically greater adverse left atrial remodeling than freedom from recurrence, but between-group differences did not remain statistically significant after correction for multiple comparisons. These findings should not be interpreted as evidence that PVI itself caused the observed changes, particularly in the absence of a non-ablated control group, and require confirmation in larger, adequately powered prospective studies incorporating continuous rhythm monitoring.

## 1. Introduction

Atrial fibrillation (AF) is the most common sustained cardiac arrhythmia and is associated with increased morbidity and mortality. Beyond its electrical substrate, AF is increasingly recognized as a manifestation of atrial cardiomyopathy, characterized by progressive structural, functional, and hemodynamic remodeling of the left atrium (LA) [1,2]. Consequently, comprehensive assessment of LA remodeling has become essential for understanding disease progression and the effects of rhythm control strategies in patients with AF. Nevertheless, in a significant number of individuals, structural remodeling may already be present before the first documented episode of AF [3].

Pulmonary vein isolation (PVI) represents a cornerstone therapy for patients with symptomatic paroxysmal and persistent AF [4]. Catheter ablation (CA) with PVI has been associated with reverse atrial remodeling in selected patients, particularly when sinus rhythm is successfully maintained, and a favorable response to the procedure is achieved [5,6,7].

Despite growing interest in echocardiographic assessment of atrial remodeling after PVI, existing studies remain heterogeneous with respect to follow-up duration, imaging methodology, and particularly the quality of the evaluated parameters [5,8,9,10,11,12,13,14,15,16].

Conventional echocardiographic parameters, including LA diameter, LA area, and LA volume index (LAVI), have been widely evaluated in previous studies and represent well-established measures for assessing both early and late structural remodeling of the LA following CA [10,17]. Speckle-tracking-derived peak atrial longitudinal strain during the reservoir phase (PALS) has emerged as a robust marker of LA mechanical function and appears to be more sensitive to dynamic functional changes, particularly during the early dynamic stages after the ablation procedure [8].

There are others, such as the LA expansion index (LA-EI) [18,19,20], LA active emptying fraction (LA-AE) [21], LA passive emptying fraction (LA-PE) [22], LA function index (LAFI) [23], LA stiffness index (LAS-si) [24], and LA kinetic energy (LAKE) [25], which have been proposed to better integrate mechanical and hemodynamic aspects of LA function. To date, only a limited number of studies have investigated these emerging parameters in the context of CA.

The aim of the present study was to perform an evaluation of LA structure and function after PVI, integrating conventional echocardiographic measurements with advanced, less validated indices of LA mechanics and hemodynamics, with particular emphasis on differences between responders and non-responders.

We hypothesized that late post-procedural assessment would reveal meaningful differences in LA mechanical function between responders and non-responders to PVI, and that advanced echocardiographic parameters would provide incremental information beyond traditional structural measures with respect to atrial remodeling and clinical outcome.

## 2. Materials and Methods

### 2.1. Study Population

This study was designed as a single-center, prospective observational cohort study conducted at Kashubian Center for Heart and Vascular Diseases in Wejherowo, Poland, between August 2023 and July 2024. The study was approved by the institutional ethical review board nr KB -34/23, and all participants provided written informed consent. We screened 51 consecutive patients who were considered suitable candidates for both cryoballoon ablation (CBA) and radiofrequency CA (RFCA), who accepted the study protocol and provided consent for further contact and follow-up. Patients were selected according to the inclusion and exclusion criteria.

### 2.2. Inclusion and Exclusion Criteria

Patients with any form of AF, including paroxysmal, persistent, and long-standing persistent AF, were eligible for inclusion provided they had maintained sinus rhythm for at least 6 weeks prior to the first study assessment. All participants were required to be ≥18 years of age and to have provided written informed consent to participate in the clinical study.

Patients were excluded from the study if they did not meet the inclusion criteria. Additional exclusion criteria included pregnancy, intracardiac thrombus precluding the procedure, and the presence of atrial fibrillation or another atrial tachyarrhythmia at the scheduled follow-up visit or any documented atrial tachyarrhythmia occurring within the preceding six weeks before the follow-up echocardiographic examination. This criterion was introduced to minimize the potential bias related to transient left atrial mechanical dysfunction (atrial stunning) following recent atrial tachyarrhythmia [26,27]. Therefore, only patients who had maintained sinus rhythm for at least six weeks before the follow-up echocardiographic assessment were included in the final remodeling analysis.

### 2.3. Echocardiography

Transthoracic echocardiography (TTE) was performed using the Vivid E95 system (GE, Milwaukee, WI, USA) to assess LA remodeling by evaluating LA size and functional parameters before and after CA. Speckle-tracking strain analysis was performed offline using EchoPAC software, version 210 (GE, Milwaukee, WI, USA). Echocardiographic parameters were assessed in accordance with the recommendations of the European Association of Cardiovascular Imaging (EACVI) by an experienced echocardiographer and were independently verified by a second physician; at the 12-month follow-up visit, this assessment was performed blinded to the patient’s arrhythmia-recurrence (responder/non-responder) status. Agreement between the two assessors was confirmed by visual comparison of the measurements rather than by a formally quantified intra- or interobserver reproducibility protocol, which represents a methodological limitation of the present study (see Limitations). Left atrial volumes were measured at end-systole (LAVmax), immediately before atrial contraction (LAVpreA) and at end-diastole (LAVmin), and were indexed to body surface area. LA functional and hemodynamic indices were derived using the following established formulas:

LA ejection volume (LAEV) = LAVmax − LAVmin.

LA ejection fraction (LAEF) = [(LAVmax − LAVmin)/LAVmax] × 100%.

LA passive emptying fraction (LA-PE) = [(LAVmax − LAVpreA)/LAVmax] × 100%.

LA active emptying fraction (LA-AE) = [(LAVpreA − LAVmin)/LAVpreA] × 100%.

LA expansion index (LA-EI) = [(LAVmax − LAVmin)/LAVmin] × 100%.

LA function index (LAFI) = (LAEF × LVOT velocity–time integral)/LA volume index at end-systole.

LA kinetic energy (LAKE) = 0.5 × ρ × LA stroke volume × (peak transmitral A-wave velocity)^2^.

LA stiffness index (LAS-si) = (E/e′)/LAS-r.

where ρ denotes blood density (1.06 g/cm^3^) and LA stroke volume equals LAVmax − LAVmin. Speckle-tracking deformation analysis was performed from apical four- and two-chamber views at frame rates of 50–80 frames/s, using the R-wave as the zero-reference point for strain measurement; accordingly, reservoir strain (LAS-r) is reported as a positive value, whereas conduit (LAS-cd) and contractile (LAS-ct) strain are reported as negative values.

Transesophageal echocardiography (TEE): In all patients, regardless of thromboembolic risk assessed by the CHA_2_DS_2_-VASc score or the type of arrhythmia, transesophageal echocardiography was performed within 24 h prior to the procedure to exclude intracardiac thrombus, particularly in the LA appendage. The examination was carried out using the same machine as in TTE. The procedure itself was conducted under mild conscious sedation, achieved with the administration of at least midazolam and/or fentanyl.

### 2.4. Anticoagulation Management

The management of oral anticoagulation during the perioperative period was conducted in the same manner and consisted of withholding the morning dose of anticoagulant medications. The procedure was performed according to standard practice, with administration of unfractionated heparin in doses adjusted to achieve an activated clotting time (ACT) of 300–350 s. The initial loading dose was 120 IU/kg, with half of the dose administered before transseptal puncture. After two transthoracic echocardiographic examinations—one performed immediately after completion of the procedure and the second 4 h later—patients were restarted on oral anticoagulant therapy according to standard dosing regimens.

### 2.5. Ablation Procedure

Both CBA and RFCA procedures were performed under mild conscious sedation using midazolam and fentanyl. Ultrasound guidance was routinely used to obtain vascular access. Access was achieved exclusively via the right femoral vein. For CBA, three venous punctures were performed to accommodate the coronary sinus catheter, His bundle catheter, and the FlexCath sheath, whereas for RFCA, three punctures were also used for placement of the coronary sinus catheter, His bundle catheter, and the Agilis steerable sheath.

CBA was performed using a third-generation ArcticFront (Medtronic, Minneapolis, MN, USA). Transseptal puncture was carried out in all patients with the use of the AcQCross transseptal system. All patients were treated with the 28 mm cryoballoon and 20 mm Achieve mapping catheter. The number of freeze applications per pulmonary vein (PV) was operator-dependent, but most patients received one (240 s) freeze application with a target time to isolation (TTI) below <70 s. PV potentials were actively sought using the Achieve mapping catheter and were successfully identified in the majority of patients. When the predefined criteria were not met, an additional 180 s bonus freeze was applied, while no other supplementary applications (such as carina or ridge ablation in the left superior PV region) were performed. Phrenic pacing and monitoring were performed during ablation over the right-sided PVs.

RFCA was started with endocardial surface reconstruction performed using a circular mapping catheter (Advisor™ Lasso FL Mapping Catheter; Abbott, Plymouth, MN, USA) for fast anatomical mapping. Ablation was performed with radiofrequency energy using a standard irrigated-tip ablation catheter (Tactiflex™, Abbott). The standard ablation settings included a preselected power (45 W,20 s) and a flow rate of 13 mL/min. At the posterior LA wall, power delivery was limited (50 W,10 s). The standard contact force ranged between 10 and 20 g. Patients presenting in AF were cardioverted. During stable sinus rhythm, the electroanatomical map was obtained. A bipolar peak-to-peak electrogram amplitude of <0.5 mV was considered indicative of low-voltage, diseased myocardial tissue. In all patients, standard ostial PVI was performed. Ablation endpoints were lack of local pace capture and bidirectional conduction block over linear lesions.

### 2.6. Follow-Up

Patients were followed up on the day of the procedure and again after 12 months. At both time points, all patients underwent a standard 12-lead ECG, TTE, and TEE. Between scheduled visits, patients were instructed to report symptoms suggestive of arrhythmia recurrence, prompting additional evaluation with a 12-lead ECG or 24 h Holter ECG, either at our center or by their referring physician. Procedural failure was defined as any documented episode of AF or AFL on a standard 12-lead ECG, or any supraventricular tachyarrhythmia lasting >30 s on 24 h Holter ECG monitoring. Patients meeting these criteria were classified as non-responders. However, only patients with AF or AFL at the scheduled follow-up visit or with a documented episode within the preceding six weeks were excluded from the echocardiographic analysis to minimize bias related to transient left atrial stunning.

### 2.7. Statistical Analysis

Analyses were conducted with IBM SPSS Statistics, version 23. Missing data were not imputed; only patients with complete follow-up and eligible for echocardiographic analysis were included in the final analysis. Continuous data are reported as the mean ± standard deviation (SD) and were compared using independent-samples Student’s *t*-tests. Categorical variables were described using numbers and percentages and compared with Fisher’s exact test. Normality of continuous variables was assessed using the Shapiro–Wilk test. Baseline clinical and echocardiographic characteristics comparisons between responders and non-responders (Table 1 and Table 2 ) were performed using independent-samples Student’s *t*-tests for continuous variables and Fisher’s exact test for categorical variables. Baseline-to-follow-up changes within each group are summarized descriptively in Table 3, alongside the formal Group × Time interaction test described below. Longitudinal differences between responders and non-responders were analyzed using a two-way mixed-design (split-plot) analysis of variance (ANOVA), with arrhythmia-recurrence status (Responders vs. Non-responders) as the between-subjects factor and time (baseline vs. follow-up) as the within-subjects (repeated-measures) factor. Because the study aimed to comprehensively screen the full range of conventional and advanced echocardiographic indices of left atrial structure and function rather than test a single predefined outcome, no individual parameter was designated as a primary endpoint; all 19 parameters were treated as a single family for the purpose of multiplicity control. The Benjamini–Hochberg false discovery rate (FDR) procedure was applied across this family; both nominal and FDR-adjusted *p*-values are reported, and all reported associations should be regarded as hypothesis-generating rather than confirmatory. Given the limited size of the non-responder subgroup (n = 10), no formal a priori sample-size calculation was performed, and all responder/non-responder comparisons are explicitly presented as exploratory.

## 3. Results

The baseline characteristics of the study population are shown in Table 1. A total of 51 patients were included. The mean age was 61 years, and 59.6% of the patients were men. Paroxysmal atrial fibrillation was present in 64% of the study population. All patients underwent PVI using one of two selected techniques: CBA or RFCA, which were performed in 62.75% and 37.25% of patients, respectively.

Follow-up at 12 months was completed in 47 patients, while 4 patients did not attend the scheduled follow-up visit (Figure 1). During follow-up, AF was documented in 5 patients, either at the time of the follow-up visit and/or within the 6 weeks preceding the visit. These patients were excluded from further analysis.

Among the 42 patients included in the final analysis, 10 patients (23.8%) experienced a recurrence of clinically documented atrial tachyarrhythmia (AF, atrial flutter, or atrial tachycardia)—these patients were classified as non-responders, whereas 32 (76.2%) patients met the criteria for a positive procedural response and were classified as responders. The mean follow-up duration was 372 ± 17 days.

Baseline demographic and clinical characteristics were largely comparable between the two groups, with no significant differences observed in age, body mass index, or the prevalence of major comorbidities. AF–related parameters, including AF type, risk scores (CHA_2_DS_2_-VASc, HAS-BLED), symptom burden, and AF history, did not differ significantly between groups. Prior rhythm control strategies, including electrical cardioversion and antiarrhythmic drug therapy, were similarly distributed. Among concomitant medications, beta-blocker use tended to be more common in responders compared with non-responders (81% vs. 50%), showing a trend toward statistical significance (*p* = 0.06), compared with non-responders, whereas other pharmacological treatments showed no significant differences. Procedural characteristics and periprocedural outcomes were comparable between groups.

Baseline echocardiographic characteristics are summarized in Table 2 and were comparable between responders and non-responders. Left ventricular ejection fraction remained stable during follow-up, with no significant difference between measurements (60.3 ± 6.92% vs. 60.8 ± 5.46%; *p* = 0.95). No significant differences were observed in LA size or volume parameters, including LA diameter, LA area, LAVI assessed by both MOD and AL methods, as well as maximal, minimal, and pre-A LA volumes. Similarly, indices of LA function, including reservoir, conduit, and contractile strain components, LA-EF, LAFI, LAKE, LA-EI, and average E/e′ ratio, did not differ significantly between groups. A trend toward a higher LA-PE fraction was observed in non-responders compared with responders; however, this difference did not reach statistical significance (28 ± 12 vs. 20 ± 12, *p* = 0.07).

Table 3 reports, for each of the 19 echocardiographic parameters, the extent to which the 12-month change differed between responders and non-responders, capturing the magnitude of change over time between groups rather than the significance of change within each group considered separately. For every parameter, the table presents the between-group difference in the baseline-to-follow-up change (Δ R−NR) with its 95% confidence interval, together with both the nominal *p*-value and a false discovery rate (FDR)-adjusted *p*-value accounting for the 19 parameters tested simultaneously. Baseline values for both groups are given in Table 2, and the trajectories of the parameters with the most pronounced differences are shown in Figure 2. Trajectories for the parameters with the most pronounced between-group differences are displayed in Figure 2. Only peak left atrial reservoir strain (LAS-R) demonstrated a discernible signal: values remained essentially unchanged among responders but declined markedly among non-responders, yielding a between-group difference of 6.36 percentage points (95% CI 2.28 to 10.44; *p* = 0.003). Following adjustment for multiple testing, however, this difference no longer attained statistical significance (adjusted *p* = 0.06). Several additional parameters—left atrial area, left atrial volume index, ejection fraction, and stiffness index—exhibited directionally consistent, albeit weaker, trends toward less favorable outcomes among non-responders, none of which attained statistical significance even prior to correction. Overall, no parameter demonstrated a statistically confirmed between-group difference following correction for multiple comparisons.

## 4. Discussion

In this study, we investigated the impact of PVI on LA structural and functional remodeling using comprehensive echocardiographic assessment at baseline and 12-month follow-up. While CA effectively controlled atrial arrhythmias in a substantial proportion of patients, our findings do not provide statistically robust evidence, after correction for multiple comparisons, that successful rhythm control was consistently accompanied by improvement in LA mechanical function. Descriptively, when the entire cohort was analyzed, most LA structural parameters showed a numerical tendency to increase after PVI, and this tendency was more pronounced among patients classified as non-responders, whereas responders showed relatively stable values over follow-up (Table 3); however, only the Group × Time interaction for LAS-R reached nominal significance, and this did not survive correction for multiple testing, so these patterns should be interpreted as hypothesis-generating trends rather than confirmed group differences.

In line with our observations, Erdei et al. [15] reported that successful CBA in patients with paroxysmal AF was associated with stabilization of LA structural parameters but did not lead to significant improvement in functional indices over 12 months. This suggests that while CA may prevent further atrial enlargement, mechanical recovery of LA function is not guaranteed and may be limited by pre-existing atrial remodeling.

Previous studies, including the Cryo-LAEF study, demonstrated preserved or improved LA ejection fraction following pulmonary vein isolation, particularly after cryoballoon ablation, in patients with paroxysmal AF during short-term follow-up [28]. In contrast, our study included a more heterogeneous population with both paroxysmal and persistent AF, larger baseline LA volumes, and a 12-month follow-up, which may partly explain the less pronounced improvement in LA functional parameters. In the study by Nagai et al. [29], patients with persistent AF who remained free of arrhythmia after RFCA demonstrated concordant structural and functional reverse remodeling, reflected by reductions in LA volume and improvements in LA strain parameters at short-term follow-up. In contrast, such parallel improvement was not observed in our cohort of responders, which may be partly explained by differences in baseline atrial substrate and clinical profile. Specifically, in our cohort of responders, patients exhibited larger baseline LA volumes (LAVI 43.67 ± 11.67 vs. 33.7 ± 10.4 mL/m^2^), were of comparable age (60.75 ± 10.95 vs. 57.7 ± 8.4 years), and carried a higher comorbidity burden, reflected in higher CHA_2_DS_2_-VASc scores (2.12 ± 1.38 vs. 0.67 ± 1.03). These characteristics are consistent with more advanced baseline atrial remodeling and atriopathy, which may have limited the extent of long-term functional recovery of the LA despite effective rhythm control.

In the DECAAF II subanalysis [6], LAVI assessed by cardiac MRI decreased significantly from 62.0 to 51.3 mL/m^2^ after ablation, with greater volume reduction observed in patients undergoing fibrosis-guided substrate modification compared with PVI alone. Although these measurements were obtained using a different imaging modality than echocardiography, limiting direct quantitative comparison, the direction and magnitude of change clearly indicate substantial reverse structural remodeling in patients with markedly enlarged atria. This supports the concept that in the presence of advanced atrial cardiomyopathy, PVI alone may be insufficient to induce optimal remodeling and that additional substrate modification may be required.

Previous studies demonstrated that increased LAS-Si is associated with advanced atrial remodeling and a higher risk of AF recurrence after PVI [30,31]. Notably, both Machino-Ohtsuka et al. [30] and Khurram et al. [31] assessed LA stiffness using indices that combined echocardiographic strain with invasively measured or estimated LA pressures, thereby capturing the mechanical properties of the atrial myocardium. In this context, the LAS-Si parameter used in our study may be interpreted as a non-invasive surrogate reflecting similar pathophysiological constructs of atrial stiffness and reduced atrial compliance. Although LAS-Si does not incorporate direct pressure measurements, it integrates deformation-based echocardiographic indices to characterize atrial mechanical behavior. To the best of our knowledge, this approach has not been previously evaluated in the setting of post-ablation atrial remodeling, thereby extending prior concepts of atrial stiffness assessment into a fully non-invasive framework.

LAFI, a composite measure integrating left atrial structure and mechanical function, has been identified as a marker of adverse atrial remodeling and is independently associated with incident AF and cardiovascular disease in the general population [32]. Furthermore, lower baseline LAFI has been shown to predict late AF recurrence following catheter ablation [23]. To the best of our knowledge, changes in LAFI following catheter ablation have not been previously reported. In our cohort, LAFI declined numerically more in non-responders than in responders (Table 3), but this difference in trajectory was not statistically significant after formal testing of the Group × Time interaction (*p* = 0.24) and should be regarded as a non-significant trend rather than a confirmed group difference.

However, when analyzing the available literature, considerable heterogeneity in reported structural and functional remodeling of the LA after CA becomes evident. These discrepancies likely reflect differences in baseline patient characteristics, atrial substrate, and disease burden rather than the ablation procedure itself. Pre-procedural factors such as LA size, duration and type of AF, age, and comorbidity profile may critically influence the extent and pattern of post-ablation atrial remodeling, potentially accounting for the variability in outcomes observed across published studies.

Available evidence suggests that baseline LA size itself has been shown to be an important predictor of AF recurrence after CA. In a cohort of patients studied with speckle-tracking echocardiography, those who experienced AF recurrence had significantly higher maximum LA volume, minimum LA volume, and LAVI at baseline compared with patients who maintained sinus rhythm, highlighting that larger atrial size is associated not only with worse functional parameters but also with a higher likelihood of post-ablation recurrence [33]. Consistent with this, meta-analytic data indicate that patients with AF recurrence following ablation have higher mean LAV and LAVI compared with patients without recurrence, supporting the role of LA enlargement as a risk modifier for arrhythmia recurrence [10].

Predicting AF recurrence after CA remains challenging due to the heterogeneous nature of patient populations and the multifactorial substrate underlying atrial remodeling. As highlighted by a systematic review of prognostic models, although LA size, AF phenotype, and age are frequently included as predictors, no model demonstrates consistent performance across cohorts, which is consistent with our observation that, in a clinically heterogeneous population with more advanced atrial disease, maintenance of sinus rhythm was not accompanied by measurable improvement in LA function [34].

Our study population included patients treated with both CBA and RFCA, which introduces the possibility of method-related bias, as different ablation technologies may exert distinct effects on atrial myocardial tissue. However, available evidence regarding the extent and nature of ablation-induced myocardial injury remains inconsistent. While some studies have suggested more extensive myocardial damage following CBA [35,36,37,38], others have reported comparable [39] or even opposing findings [40] when compared with RFCA. Consequently, the differential impact of ablation modality on atrial structural and functional remodeling remains uncertain, making it difficult to attribute observed remodeling patterns to the ablation technique alone. Overall, our findings are consistent with several studies reporting limited or absent improvement in LA mechanical function after PVI, while differing from other reports demonstrating reverse atrial remodeling following ablation. These discrepancies likely reflect differences in patient populations and the complex, multifactorial nature of LA remodeling, which may be influenced by disease stage, comorbidity burden, and baseline atrial substrate. Available evidence suggests that catheter ablation, using either CBA or RFCA, may favorably influence atrial remodeling or attenuate its progression; however, the magnitude of this effect is likely influenced by the degree of pre-existing atrial cardiomyopathy and successful maintenance of sinus rhythm. In our exploratory cohort, patients with recurrent arrhythmia showed numerically greater adverse remodeling than those remaining free of recurrence, although this difference was not statistically confirmed; whether baseline atrial disease severity modifies this relationship was not formally tested and remains a question for adequately powered studies. Future randomized studies with comprehensive rhythm monitoring and detailed phenotyping of atrial substrate are warranted to validate these findings and better identify patients most likely to benefit from catheter ablation. Further advances in ablation technologies, including pulsed field ablation, together with improvements in mapping systems and a better understanding of AF pathophysiology, may further optimize patient selection and long-term outcomes.

Regarding the clinical relevance of these findings, the direction of change observed in non-responders—declining PALS and LAEF alongside rising LAVI and LAS-si—mirrors the pattern previously linked to elevated filling pressures, reduced atrial compliance, and heightened arrhythmia recurrence risk [23,30,31]. Even though these between-group differences did not survive correction for multiple comparisons in our cohort, their consistent direction across several mechanistically related indices suggests they may represent a clinically meaningful signal warranting confirmation rather than a purely statistical artifact. We also note that non-responders already exhibited numerically larger LA volumes and strain values at baseline (Table 2) despite the absence of statistically significant between-group differences; given the small non-responder subgroup (n = 10), these nonsignificant baseline trends may reflect insufficient statistical power rather than true baseline equivalence, and this possibility cannot be excluded. Finally, whether responder status is independently associated with remodeling after accounting for age, AF type and burden, and baseline atrial size could only be established in a multivariable model, which the size of our non-responder subgroup did not permit; these univariate comparisons therefore require re-examination in adequately powered cohorts.

## 5. Limitations

This study has several important limitations. The small sample—in particular the 10 non-responders—and the observational, non-randomized design limit statistical power and preclude causal inference; no a priori power calculation was performed, and no individual parameter was pre-specified as a primary endpoint, since the study was designed as a broad screen of conventional and advanced left atrial indices rather than a confirmatory test of a single outcome. All responder/non-responder comparisons should therefore be regarded as exploratory and hypothesis-generating, and require validation in larger, adequately powered studies. Critically, in the absence of a non-ablated control group, changes over time cannot be attributed to PVI itself. Arrhythmia recurrence was assessed by symptom-triggered 12-lead ECG or 24 h Holter monitoring rather than continuous rhythm monitoring in every patient, which may have led to underdetection of asymptomatic recurrences and misclassification of some non-responders as responders, biasing our comparisons toward the null. The exclusion of five patients with arrhythmia at or shortly before the follow-up visit, although intended to avoid confounding by transient atrial stunning, introduces a potential selection bias; echocardiographic data permitting a sensitivity analysis in these patients were not available. The four patients (approximately 10% of the cohort) lost to follow-up were not formally compared with those completing the study, and undetected baseline differences cannot be excluded. All analyses were univariate: atrial remodeling is influenced by age, AF type and burden, comorbidities and baseline LA size, but the small non-responder subgroup precluded reliable multivariable adjustment—which we state explicitly rather than assume an absence of confounding. Population heterogeneity—paroxysmal, persistent and long-standing persistent AF, two ablation modalities, and a single-center design—limits generalizability and may itself have contributed to variability in remodeling patterns. Changes in antiarrhythmic or beta-blocker therapy during follow-up were not systematically recorded and cannot be excluded as confounders. Finally, although the 12-month assessment was performed blinded to responder status, intra- and interobserver reproducibility was not formally quantified.

## 6. Conclusions

This exploratory, hypothesis-generating study found no statistically robust evidence, after appropriate correction for multiple comparisons, that left atrial mechanical function consistently improved following PVI at 12-month follow-up. Recurrent atrial arrhythmia (non-responder status) was numerically, but not significantly after correction, associated with a pattern of unfavorable left atrial remodeling—including decline in reservoir strain and ejection fraction alongside increases in volume index and stiffness index—whereas patients free of recurrence showed relatively stable left atrial structure and function. Given the small sample size, the absence of a non-ablated control group, and the lack of continuous rhythm monitoring, these findings should not be interpreted as evidence that PVI itself caused the observed changes, nor as a confirmed difference between groups; rather, they support the hypothesis that recurrent arrhythmia is associated with unfavorable atrial remodeling, a hypothesis that requires validation in larger, adequately powered, prospective studies incorporating continuous rhythm monitoring and a control group. Emerging echocardiographic indices, including LAS-si and LAFI, may represent promising exploratory markers of post-ablation atrial remodeling, but their clinical value remains unproven and requires confirmation before any clinical application. Improved patient phenotyping may help identify individuals most likely to benefit from catheter ablation, pending validation of these hypothesis-generating findings.

## Figures and Tables

**Figure 1:**
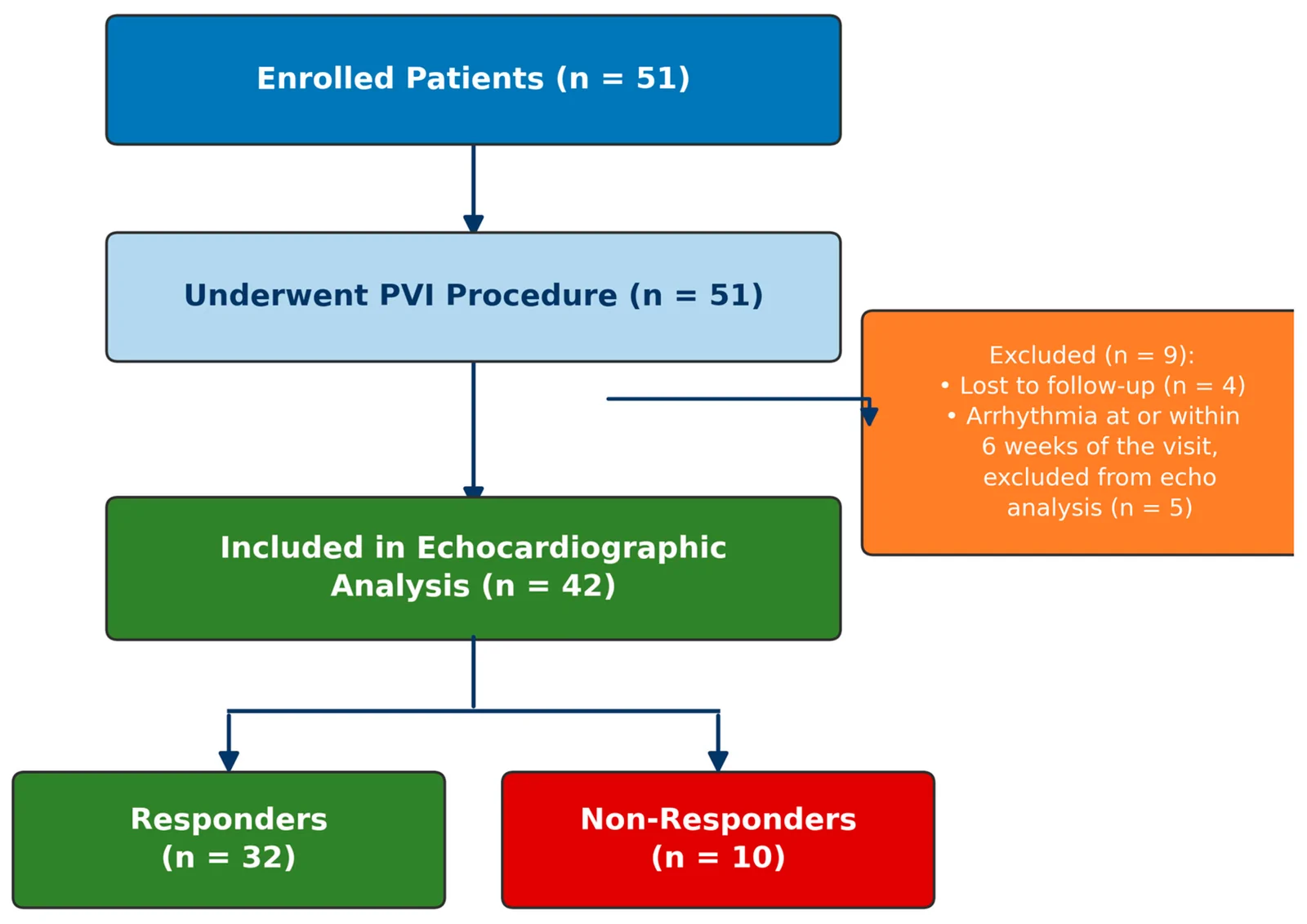
Participant flow diagram. Of 51 patients enrolled and undergoing pulmonary vein isolation (PVI), 4 were lost to follow-up, and 5 were excluded from echocardiographic analysis because of documented arrhythmia at or within 6 weeks of the 12-month visit (to avoid confounding by transient left atrial stunning). The remaining 42 patients constituted the echocardiographic analysis set and were classified as responders (n = 32) or non-responders (n = 10) according to freedom from documented atrial tachyarrhythmia recurrence.

**Figure 2:**
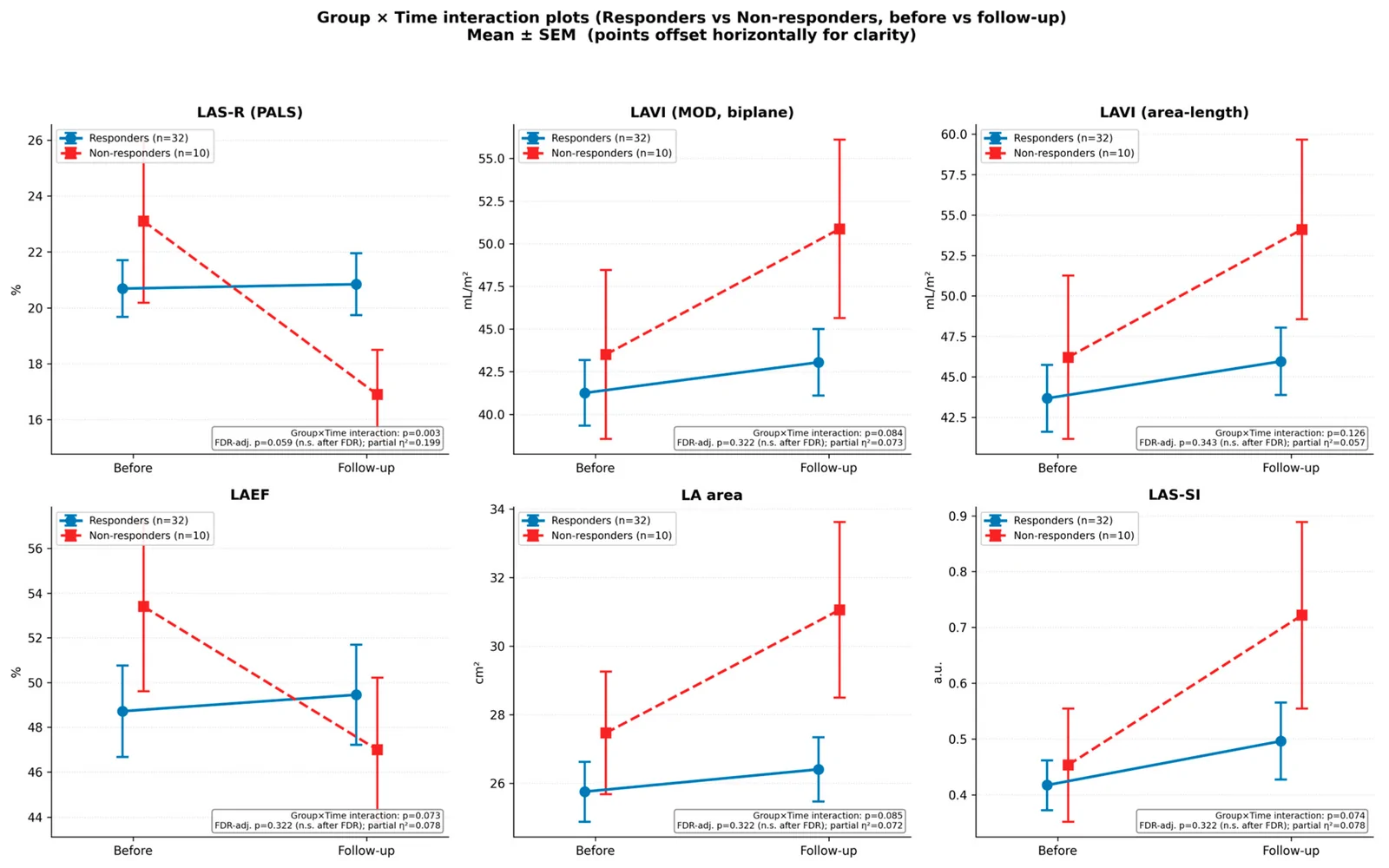
Group × Time interaction plots for six representative left atrial echocardiographic parameters spanning atrial size, volume, emptying and deformation (full results for all 19 parameters are given in Table 3). Data are mean ± SEM at baseline (Before) and 12-month follow-up (Follow-up) for responders (n = 32) and non-responders (n = 10). Annotations show the interaction *p*-value, the FDR-adjusted *p*-value (corrected across all 19 parameters), and partial η^2^; exact between-group differences with 95% confidence intervals are given in Table 3. Only the interaction for LAS-R (PALS) reached nominal significance (*p* = 0.003); it did not survive FDR correction (*p* = 0.059) and is presented as hypothesis-generating. None of the six parameters shown remained significant after correction for multiple comparisons. LAS-SI is dimensionless (a.u., arbitrary units).

**Table 1:** Baseline clinical characteristics.

	Responders	Non-Responders	*p*-Value
Demographics			
Age (years, mean ± SD)	60.75 ± 10.95	61.2 ± 9.16	0.46
Sex (male, n, n%)	22/68.75	5/50	0.06
BMI (kg/m^2^, mean ± SD)	30.35 ± 4.96	28.17 ± 6.21	0.30
Comorbidities			
Heart Failure (n, n%)	9/28	1/10	0.40
Chronic Coronary Syndrome (n, n%)	3/9	1/10	1.00
Chronic Kidney Disease (n, n%)	5/15.6	0/0	0.31
Hypertension (n, n%)	23/71.8	8/80	1.00
Diabetes (n, n%)	6/18.7	1/10	1.00
Obesity BMI >30 kg/m^2^ (n, n%)	13/40.6	5/50	0.72
Atrial fibrillation characteristic			
PAF (n, n%)	22/68.7	9/90	0.24
CHA2DS2-VASc score (mean ± SD)	2.12 ± 1.38	1.9 ± 0.87	0.55
HAS-BLED score (mean ± SD)	0.68 ± 0.69	0.8 ± 0.63	0.64
EHRA score (mean ± SD)	2.68 ± 0.64	2.8 ± 0.79	0.69
ECV in history (mean ± SD)	1.25 ± 1.79	0.6 ± 0.84	0.12
AAD in history (mean ± SD)	0.56 ± 0.62	0.9 ± 0.87	0.28
History of AF (years, mean ± SD)	5.51 ± 4.45	4.4 ± 3.4	0.41
Drugs			
B-blockers (n, n%)	26/81	5/50	0.06
AAD type I (n, n%)	4/12.5	4/40	0.07
AAD type III (n, n%)	6/18.75	1/10	1.00
DOAC (n, n%)	29/90	10/100	1.00
Apixaban (n, n%)	12/37.5	4/40	1.00
Rivaroxaban (n, n%)	16/50	4/40	0.72
Dabigatran (n, n%)	1/3.1	2/20	0.14
Vitamin K Antagonist (n, n%)	0/0	0/0	1.00
ACE-I (n, n%)	17/53	4/40	0.72
Ablation procedure			
Cryoablation (n, n%)	24/75	5/50	0.28
Radiofrequency ablation (n, n%)	8/25	5/50	0.24
Day of procedure (day, mean ± SD)	1.69 ± 0.47	1.7 ± 0.48	0.96
Duration of hospitalization (days, mean ± SD)	2.8 ± 1.09	2.8 ± 0.62	0.85

Baseline demographic and clinical characteristics of the study population undergoing pulmonary vein isolation. Data include age, sex, body mass index, cardiovascular comorbidities, type of atrial fibrillation, and pharmacological treatment prior to ablation. Continuous variables are described in terms of means and standard deviations, while categorical data are described using percentages and frequencies. Abbreviations: AF—atrial fibrillation; PAF—paroxysmal atrial fibrillation; BMI—body mass index; EHRA—European Heart Rhythm Association symptom score; ECV—electrical cardioversion; AAD—antiarrhythmic drug; OAC—oral anticoagulation; DOAC—direct oral anticoagulant; VKA—vitamin K antagonist; ACE-I—angiotensin-converting enzyme inhibitor.

**Table 2:** Echocardiographic characteristics at baseline between responders and non-responders.

	Responders	Non-Responders	*p*-Value
LA diameter [mm]	42.56 ± 6.38	44.70 ± 4.81	0.34
LA area [cm^2^]	25.76 ± 4.92	27.47 ± 5.66	0.36
LAVI (MOD) [mL/m^2^]	41.25 ± 10.88	43.51 ± 15.67	0.61
LAVI (AL) [mL/m^2^]	43.67 ± 11.67	46.20 ± 15.99	0.59
LAS-R [%]	20.69 ± 5.72	23.10 ± 9.22	0.32
LAS-CD [%]	−9.72 ± 3.96	−12.60 ± 6.62	0.10
LAS–CT [%]	−10.62 ± 3.70	−10.30 ± 3.83	0.81
LAEF [%]	48.72 ± 11.58	53.40 ± 11.96	0.27
LAEV [mL]	30.28 ± 8.88	33.90 ± 10.68	0.29
LAV max [mL]	65.81 ± 19.87	65.90 ± 24.17	0.99
LAV min [mL]	35.19 ± 17.23	32.10 ± 17.94	0.63
LAVpreA [mL]	53.62 ± 20.36	48.90 ± 22.83	0.54
LAKE [kdyn.cm]	82.0 ± 77.1	68.5 ± 48.7	0.61
LAFI	25.73 ± 13.16	30.42 ± 15.18	0.35
LA Expansion Index [%]	105 ± 51	129 ± 67	0.22
LA AE [%]	35 ± 13	36 ± 12	0.92
LA PE [%]	20 ± 12	28 ± 12	0.07
LAS-si	0.42 ± 0.25	0.45 ± 0.32	0.71
E/e’ avr	7.63 ± 3.05	8.57 ± 3.18	0.40

Echocardiographic parameters of the entire study population assessed before pulmonary vein isolation stratified to responders and non-responders. Abbreviations: LA—left atrium; LAVI—left atrial volume index; MOD—biplane method of disks (modified Simpson’s rule); AL—area-length method; LAVmax—maximal left atrial volume; LAVmin—minimal left atrial volume; LAVpreA—left atrial volume before atrial contraction; LAEV—left atrial ejection volume; LAS-r—left atrial reservoir strain; LAS-cd—left atrial conduit strain; LAS-ct—left atrial contractile strain; LAS-si—left atrial stiffness index; LA EF—left atrial ejection fraction; LA PE—left atrial passive emptying fraction; LA AE—left atrial active emptying fraction; LAFI—left atrial functional index; E/e′—ratio of early mitral inflow velocity to early diastolic mitral annular velocity; a.u.—arbitrary units (LAS-si is a dimensionless ratio, calculated as E/e′ divided by LAS-r, and therefore has no physical unit); n.s—not significant.

**Table 3:** Between-group difference in 12-month change (Group × Time interaction) for 19 echocardiographic parameters, comparing responders and non-responders.

Parameter	Δ (R−NR) [95% CI]	*p*	p (FDR)
LAS-R [%]	6.36 [2.28, 10.44]	0.003	0.059
LAEF [%]	7.13 [−0.71, 14.98]	0.073	0.322
LAS-si	−0.19 [−0.40, 0.02]	0.074	0.322
LAVI (MOD) [mL/m^2^]	−5.57 [−11.92, 0.78]	0.084	0.322
LA area [cm^2^]	−2.94 [−6.30, 0.42]	0.085	0.322
LA AE [%]	8.67 [−2.46, 19.80]	0.123	0.343
LAVI (AL) [mL/m^2^]	−5.62 [−12.89, 1.65]	0.126	0.343
LA Expansion Index [%]	22.81 [−10.59, 56.21]	0.175	0.380
LAS-CT [%]	−2.42 [−6.17, 1.34]	0.201	0.380
LAVmax [mL]	−9.15 [−23.91, 5.61]	0.218	0.380
LAVmin [mL]	−7.21 [−19.17, 4.76]	0.231	0.380
LAFI	5.30 [−3.68, 14.29]	0.240	0.380
LAS-CD [%]	−2.22 [−6.33, 1.90]	0.282	0.413
LAVpreA [mL]	−5.56 [−18.94, 7.83]	0.406	0.551
LA diameter [mm]	−1.11 [−4.07, 1.84]	0.452	0.572
E/e’ avr	−0.61 [−3.17, 1.95]	0.633	0.720
LA PE [%]	−1.87 [−9.97, 6.24]	0.644	0.720
LAEV [mL]	−0.89 [−7.37, 5.58]	0.782	0.825
LAKE [kdyn.cm]	4.9 [−40.5, 50.3]	0.827	0.827

R = Responders (n = 32); NR = Non-responders (n = 10). Δ (R−NR) = difference between responders and non-responders in the mean change from baseline to 12-month follow-up, with 95% CI; a positive value indicates a more favorable change in responders. Baseline values for both groups are reported in Table 2. *p* = *p*-value for the between-group difference; p (FDR) = the same *p*-value adjusted for testing 19 parameters. Parameters are sorted by ascending p. Only LAS-R attains *p* < 0.05 prior to adjustment (underlined); none remain significant following adjustment—findings should be regarded as exploratory/hypothesis-generating given the limited non-responder subgroup (n = 10). Abbreviations: LA—left atrium; LAVI—left atrial volume index; MOD—biplane method of disks (modified Simpson’s rule); AL—area-length method; LAVmax—maximal left atrial volume; LAVmin—minimal left atrial volume; LAVpreA—left atrial volume before atrial contraction; LAEV—left atrial ejection volume; LAS-R—left atrial reservoir strain; LAS-CD—left atrial conduit strain; LAS-CT—left atrial contractile strain; LAS-si—left atrial stiffness index; LAEF—left atrial ejection fraction; LA PE—left atrial passive emptying fraction; LA AE—left atrial active emptying fraction; LAFI—left atrial functional index; LAKE—left atrial kinetic energy; E/e′—ratio of early mitral inflow velocity to early diastolic mitral annular velocity; a.u.—arbitrary units (LAS-si and LAFI is a dimensionless ratio- has no physical unit).

## Data Availability

The data supporting the report are available from Jonasz Kozielski (jonaszkozielski@gmail.com) on reasonable request.

## Abbreviations

The following abbreviations are used in this manuscript:

AF	Atrial fibrillation
LA	Left atrium
RFCA	Radiofrequency catheter ablation
CBA	Cryoablation
